# Consumption of meat and dairy substitute products amongst vegans, vegetarians and pescatarians

**DOI:** 10.29219/fnr.v67.9081

**Published:** 2023-03-31

**Authors:** Live Edvardsen Tonheim, Synne Groufh-Jacobsen, Tonje Holte Stea, Sigrun Henjum

**Affiliations:** 1 Department of Nursing and Health Promotion, Faculty of Health Sciences, Oslo Metropolitan University, Oslo, Norway; 2 Department of Nutrition and Public Health, Faculty of Health and Sport Sciences, University of Agder, Kristiansand, Norway; 3 Department of Health and Nursing Sciences, Faculty of Health and Sport Sciences, University of Agder, Kristiansand, Norway

**Keywords:** plant-based substitutes, plant-based diet, dietary intake, macronutrients, salt, vegans and vegetarians

## Abstract

**Background:**

An increasing number of people adhere to plant-based diets, and the market for plant-based meat and dairy substitute products has been expanding rapidly.

**Objective:**

To examine total intake of macronutrients and salt in a sample of Norwegian vegans, vegetarians and pescatarians; the consumption frequency of plant-based meat and dairy substitutes and raw ingredients used in these products; and the contribution to total macronutrient and salt intake from these products.

**Design:**

A cross-sectional design using single 24-h dietary recall to assess the intake of macronutrients, salt and substitute products that the participants (*n* = 158 Norway residents [age 18–60 years]: vegans [*n* = 83]; vegetarians [*n* = 47]; pescatarians [*n* = 28]) consumed. The chi-square test with pairwise comparisons and the Kruskal-Wallis test with post hoc test were used to compare differences between diet groups. Macronutrient and salt intake were assessed relative to the Nordic Nutrition Recommendations (NNR).

**Results:**

Dietary macronutrient intake fell within NNR recommendations, with a favourable distribution of fatty acids and high levels of dietary fibre. Most of the vegans (90%), vegetarians (68%) and pescatarians (64%) consumed meat or dairy substitutes. The main raw ingredient in the substitute products was soy, followed by oats and peas. Overall, substitute products contributed to 12% of total energy and 16% of total salt intake. The substitute products contributed to higher saturated fatty acid (SFA) intake amongst vegans (27% of total SFA intake) compared with vegetarians (10%) and pescatarians (8%). Moreover, substitute products contributed to higher protein intake in vegans (19%) compared with pescatarians (7%).

**Conclusion:**

Most participants consumed meat or dairy substitute products, suggesting that these products are included regularly in Norwegian plant-based diets. Furthermore, substitute products may contribute to dietary fat, SFA and protein intake amongst vegans.

## Popular scientific summary

- Most of the vegans (90%), vegetarians (68%) and pescatarians (64%) consumed meat or dairy substitutes.
- The main raw ingredient in the substitute products was soy, followed by oats and peas.
- Overall, substitute products contributed to 12% of total energy and 16% of total salt intake in participants’ diets.
- Dietary macronutrient intake fell within NNR recommendations, with a favourable distribution of fatty acids and high levels of dietary fibre.

Plant-based diets have been gaining popularity rapidly in the Western world (1), with their prevalence in Europe and the United States estimated to be between 1 and 8% (2, 3). In Norway, it is estimated that approximately 1% of the population adheres to a vegan diet, 4% to a vegetarian diet and 7% to a flexitarian or semi-vegetarian diet (4). In keeping with this trend, sales of plant-based substitute products for meat and dairy have grown significantly (5, 6).

Multiple studies have examined nutrient intake associated with the consumption of plant-based diets (7–9), and adhering to such diets generally is associated with health benefits and reduced risk of several chronic diseases (10–12). Traditionally, plant-based diets contain ample amounts of vegetables, legumes, whole grains, nuts and seeds (13), which contribute to a beneficial nutrient profile comprising low levels of saturated fat and salt, and high levels of dietary fibre and polyunsaturated fat (13, 14).

Today, a wide selection of plant-based products intended to mimic the function, taste and texture of meat and dairy is readily available (5, 6). However, previous studies suggest that wide variations in nutritional quality exist between and within different categories of these products (15–19). However, knowledge about meat and dairy substitutes’ nutritional contribution to the diet is limited (20). Although raw ingredients in plant-based substitutes – for example, soy, oats and various legumes – are associated with beneficial health effects, this may not necessarily apply to the final meat or dairy substitute products (21). During food processing, nutrients – for example, vitamins, minerals and trace elements – may be lost (16), and less-healthy ingredients – for example, salt, sugar and saturated fats – may be added, altering the final product’s nutrient value (22). Furthermore, high consumption of meat and dairy substitutes has been thought to contribute to the rise in consumption of ultra-processed foods in plant-based diets (23).

Although vegans, vegetarians and pescatarians are likely to consume more meat and dairy substitute products than omnivores, few existing studies have examined substitute consumption based on adherence to different plant-based diets (7, 24, 25). Furthermore, studies that assess contributions to the intake of macronutrients and salt and raw ingredients from meat and dairy substitute products also are lacking. In a recently published study investigating dietary habits amongst Norwegians adhering to different types of plant-based diets, 49% of the vegans, 33% of the vegetarians and 32% of the pescatarians reported daily consumption of dairy substitutes (26), and 25% reported weekly consumption of meat substitutes. However, this study only assessed consumption frequency, so the consumption of these products needs to be examined in more detail.

Thus, the present study aims to examine the total intake of macronutrients and salt in a sample of Norwegian vegans, vegetarians and pescatarians; consumption frequency of plant-based meat and dairy substitutes and raw ingredients used in these products; and the contribution to dietary macronutrient and salt intake from these products.

## Methods

This cross-sectional study included 158 participants comprising vegans (*n* = 83), vegetarians (*n* = 47) and pescatarians (*n* = 28) living in the eastern part of Norway, ranging in age between 18 and 60. The participants were recruited through a previous study assessing iodine status in vegans, vegetarians and pescatarians (27), with convenience and snowball sampling methods used in recruiting. Participants who provided a written consent to be contacted in the prior study, by Groufh-Jacobsen, received an invitation to participate in this study by text message. Altogether, 166 subjects out of 192 invited agreed to participate, and 161 completed the study, with a participation rate of 84%. Furthermore, three participants were excluded during data analysis due to meat consumption or fasting, leaving a final total of 158.

Classification into the respective diet groups was based on foods included in the diet as assessed through an electronic questionnaire administered in the previous study by Groufh-Jacobsen. Participants who answered ‘never’ when asked whether they eat animal source foods were classified as vegans. Participants who reported not consuming meat/meat products/poultry/fish/fish products, but said they consume milk and/or dairy products and/or eggs ‘seldom’, ‘sometimes’ or ‘often’, often classified as lacto-ovo vegetarians, were classified as vegetarians. Finally, those who reported consuming fish or shellfish but not meat/meat products/poultry were classified as pescatarians. Classification to dietary groups was confirmed based on the data collected through 24-h dietary recalls, with the exception of two participants who were reclassified accordingly. The electronic questionnaire also was used to collect background information (e.g. age, height, weight, marital status, education level, country of origin and smoking habits).

### Data collection

Between January and June 2020, single 24-h dietary recalls (24 h) were conducted. One 24 h was performed via phone using semi-structured interviews (28). To ensure recording of dietary intake on both workdays and weekend days, the 24 h were conducted on different weekdays. 77% of recalls reported workday dietary intake, and there was no difference between the groups. The same researcher conducted all interviews, and the information was logged into written records consecutively.

In the 24 h, participants were asked to list all foods, beverages and supplements consumed throughout the previous day in detail. They also were asked to provide descriptions of quantity measures as indicated through serving methods – for example, sizes of glasses, bowls and plates – in addition to the size and share of the whole product, portion or recipe; number of pieces/products; intake in centimetres of product with foods such as cucumbers or celery; and in some cases, quantities in grams. Participants were prompted to describe all items in terms of product type or brand, whether the food was organic, preparation methods and each meal’s fat and sugar content, for example, percentages of bread and/or pasta in the whole meal. Participants also were asked to list all ingredients in composite foods or dishes, and if possible, also include quantities. In cases in which a specific recipe was used, online searches for the recipe were conducted, so that the participants could confirm the recipe’s accuracy. The participants subsequently were asked whether they had made any changes to the recipe in quantity or type of ingredients, and to describe consumed quantity in terms of share of the total recipe. During the final stages of the interview, probing questions were used to help the participants remember details.

### Data coding

All reported food items were assigned a food code corresponding to a food or beverage registered in the Norwegian Food Composition Table (29). In cases in which no comparable food or beverage could be found in the table, the Swedish Food Composition Database (Livsmedelsdatabasen) (30) or Dutch Food Composition Database (NEVO) (31) was used and applied to products such as hemp seeds, goji berries, psyllium husk and sweet chili sauce. If the food item was not available in either of the databases, the nutritional content was recorded manually from the package label.

All food items and beverages that each participant consumed during the 24 h survey period were converted into grams of edible portions. To standardise nutrient calculations, an online diet registration tool (Kostholdsplanleggeren) (32) and a booklet guide, both based on the Norwegian Food Composition Table (33), were used. Whenever a product’s quantity was described in a measure other than that found in the online diet registration tool or booklet guide, the following conversions were applied: 1 decilitre = 7 tablespoons; 1 tablespoon = 3 teaspoons. In cases in which the participant described quantity as a share of a product but did not know the product’s total weight, amount or size, this information was obtained from the producer’s website, online stores or the physical product’s packaging. If no information on food items’ quantity or weight was available, one of the researchers weighed the food for an approximate calculation. The mean of two weightings was used for each product, and the same researcher weighed all products on the same kitchen scale. Weighting was applied to products such as different dried berries and fruits, coconut flakes and different types of plant-based spreads.

Standardised recipes were developed for composite foods and dishes that were not described in sufficient detail to identify all ingredients and intake quantity. This applied to most of the takeaway or restaurant dishes, as well as the vegetarian or vegan versions of foods or dishes traditionally made with meat, dairy or eggs, for example, different baked goods, pizzas, pancakes, waffles, vegan spring rolls and kebabs.

### Classification of meat and dairy substitutes

Substitute products mimicking meat or dairy products were defined as meat or dairy substitutes. The definition did not include tofu or seitan in its original form, but more highly processed products using these ingredients, for example, patés made from tofu or sausages made from seitan. For purposes of describing raw ingredients based on product categories, all meat and dairy substitutes were classified based on the product category that the substitute intended to mimic.

### Calculation of energy, total macronutrient and salt intake, and raw ingredients

Total dietary intake and contribution to total dietary intake of energy, macronutrients and salt from substitute products were calculated using ‘FoodCalc’ (34), a programme that utilises food composition tables to calculate per-person nutrient intake based on recorded food intake (food code and amount in grams). Information about raw ingredients was obtained from the manufacturer’s website if available, or else manually from online grocery stores, and used to classify products based on raw ingredients. The non-water ingredient with the highest contribution by percentage was defined as the main raw ingredient.

### Ethical approval

The Norwegian Centre for Research Data/NSD/101332 and the Regional Committees for Medical and Health Research Ethics, 2019/653/REC Southeast, approved this study. Written and oral information about the study was provided to participants, and a written consent was obtained from participants before the study began.

### Statistical analysis

Statistical analyses were conducted in IBM SPSS (Versions 27 and 28) (IBM Corp., Armonk, NY, USA). The data’s normality was examined using the Shapiro-Wilk’s test and a visual examination of QQ plots and histograms. Normally distributed data were presented as mean ± standard deviation (SD) and non-normally distributed data as median and 25th and 75th percentiles (p25, p75). A chi-square test with pairwise comparisons was used to compare differences between the diet groups using categorical variables (gender, marital status, education level, work status, country of birth, smoking habits, supplement use (yes/no) and reported use of meat and dairy substitutes). A Kruskal-Wallis test with post hoc test was used to examine differences in macronutrient and salt intake, as well as differences in contribution to macronutrient and salt intake from plant-based substitutes between the diet groups using non-parametric, continuous variables. A significance level of 0.05 was applied in all tests.

## Results

Table 1 outlines the key characteristics of the participating vegans (*n* = 83), vegetarians (*n* = 47) and pescatarians (*n* = 28). In all diet groups, a large number of participants were women, had more than 12 years of education and were non-smokers. The diet groups did not differ significantly in age, education level, work status, ethnicity, diet duration, use of supplements, smoking habits and body mass index (BMI).

**Table 1:** Background characteristics of participating vegans (*n* = 83), vegetarians (*n* = 47) and pescatarians (*n* = 28)

Background		Total	Vegans	Vegetarians	Pescatarians	*P*-value^1^
Participants, *n* (%)		158 (100)	83 (53)	47 (30)	28 (18)	
Gender	Women, *n* (%)	117 (74)	54 (65)^a^	37 (79)^b^	26 (93)^ab^	0.010*
	Men, *n* (%)	41 (26)	29 (35)^a^	10 (21)^b^	2 (7)^ab^	
Age in years, mean ± SD		30 ± 9	31 ± 9	31 ± 10	29 ± 9	0.266
BMI (kg/m^2^), mean ± SD		23.1 ± 3.5	22.8 ± 2.8	23.8 ± 4.7	23.1 ± 3.0	0.789
Country of birth	Norway, *n* (%)	133 (84)	71 (86)	38 (81)	24 (86)	0.757
	Other, *n* (%)^2^	25 (16)	12 (14)	9 (19)	4 (14)	
Marital status	Single, *n* (%)	79 (50)	37 (45)	25 (53)	17 (61)	0.290
	Cohabitant/Married, *n* (%)	79 (50)	46 (55)	22 (47)	11 (39)	
Education level	≤12 years, *n* (%)	28 (18)	15 (18)	9 (19)	4 (14)	0.860
	>12 years, *n* (%)	130 (82)	68 (82)	38 (81)	24 (86)	
Work status	Unemployed, *n* (%)	6 (4)	4 (5)	1 (2)	1 (4)	0.860
	Student, *n* (%)	50 (32)	27 (33)	13 (28)	10 (36)	
	Employed, *n* (%)	102 (65)	52 (63)	33 (70)	17 (61)	
Adherence to diet in years, mean ± SD		5 ± 3	4 ± 3	6 ± 4	5 ± 4	0.187
Consumption of dietary supplements, *n* (%)^3^		109 (69)	62 (75)	29 (62)	18 (64)	0.257
Cigarette smoker, *n* (%)		14 (9)	7 (8)	6 (13)	1 (4)	0.391

1 Tested for differences between the diet groups using chi-square and Kruskal-Wallis tests; significance level used < 0.05. Marked as *.

2 Other include Colombia, Ethiopia, Germany, Great Britain, Iraq, Netherlands, Nicaragua, Poland, Portugal, Russia, Slovenia, Sweden, Ukraine and the United States.

3 Consumption of dietary supplements assessed by 24 h.

ab Diet groups with the same superscripts have proportions that differed significantly in the post hoc test, significance level used < 0.05.

### Total energy, macronutrient and salt intake

Table 2 presents the median (p25, p75) intake of macronutrients in energy percentage (E%) and salt in grams (g). All diet groups had a median intake of macronutrients in compliance with the Nordic Nutrition Recommendations (NNR), except n-3 fatty acids, which were lower than recommended in vegans and vegetarians, and salt, which was higher than recommended in pescatarians (35) (Table 2). Overall, the participants had a median (p25, p75) energy intake of 2,052 kcal (1,546, 2,516), and energy intake did not differ between groups (Table 2).

**Table 2:** Median (p25, p75) total dietary intake of macronutrients (E%) in vegans, vegetarians and pescatarians reported in 24 h

Dietary factor	Total (*n* = 158)		Vegans (*n* = 83)		Vegetarians (*n* = 47)		Pescatarians (*n* = 28)		*P*-value^1^	RI^2^
	Median	(p25, p75)	Median	(p25, p75)	Median	(p25, p75)	Median	(p25, p75)		
Kilocalories	2,052	(1,546, 2,516)	2,076	(1,521, 2,683)	2,056	(1,588–2,467)	1,910	(1,490, 2,389)	0.763	-
MJ/d	8.6	(6.4, 10.5)	8.7	(6.4, 11.2)	8.6	(6.4, 10.3)	8.0	(6.3, 10.0)	0.831	**
Fat (E%)	32.8	(26.0, 38.8)	31.3	(25.1, 37.9)	34.7	(26.7, 40.1)	33.3	(26.3, 43.6)	0.295	25–40
SFA (E%)	7.6	(4.9, 11.5)	5.8^ab^	(4.5, 8.7)	9.7^a^	(6.5, 14.8)	9.6^a^	(5.4, 14.1)	0.010*	<10
TFA (E%)	0.0	(0.0, 0.1)	0.0^ab^	(0.0, 0.0)	0.1^a^	(0.0, 0.3)	0.2^b^	(0.0, 0.3)	0.010*	***
MUFA (E%)	11.5	(8.3, 15.0)	11.1	(7.8, 15.0)	11.6	(8.6, 14.2)	12.2	(9.1, 16.1)	0.341	10–20
PUFA (E%)	7.1	(5.1, 9.9)	7.8^a^	(6.2, 10.3)	5.8^a^	(4.7, 8.0)	6.7	(5.1, 9.4)	0.030*	5–10
n-3 (E%)	0.7	(0.5–1.2)	0.7^b^	(0.5, 1.2)	0.5^a^	(0.4, 1.0)	1.2^ab^	(0.7, 1.8)	0.010*	≥1
Protein (E%)	13.6	(11.4, 15.5)	13.3^b^	(11.5, 15.1)	13.2^a^	(10.3, 15.2)	14.9^ab^	(13.0, 16.9)	0.045*	10–20
Carbohydrates (E%)	50.0	(43.4, 55.3)	50.5	(44.5, 56.2)	49.9	(41.3, 55.7)	46.9	(38.5, 51.2)	0.129	45–60
Added sugars (E%)	2.3	(0.7, 6.2)	2.0^a^	(0.3, 4.9)	4.1^ab^	(1.1, 8.4)	1.8^b^	(0.8, 4.6)	0.032*	<10
Dietary fibre (g/MJ)	4.4	(3.5, 5.7)	5.2^ab^	(4.1, 6.0)	3.8^a^	(2.8, 4.7)	4.2^b^	(2.7, 5.1)	0.010*	>3
Salt (g)	5.8	(3.5, 8.7)	6.0	(3.6, 9.9)	5.2	(3.4, 8.1)	7.0	(3.4, 8.4)	0.509	≤6

1 Tested for difference between different diet groups, Kruskal-Wallis test, significance level used: < 0.05, marked as *.

2 Recommended intakes from Nordic Nutrition Recommendations.

ab Diet groups with the same superscripts differed significantly in the post hoc test, significance level used < 0.05.

** Dependent on sex, age, weight, height and physical activity.

*** As low as possible.

#### Saturated fatty acids, polyunsaturated fatty acids and n-3 fatty acids

Vegans reported lower median (p25, p75) consumption of saturated fatty acids (SFAs) (5.8 E%, 4.5, 8.7) than both vegetarians (9.7 E%, 6.5, 14.8) (*P* < 0.001) and pescatarians (9.6 E%, 5.4, 14.1) (*P* = 0.002) (Table 2). Furthermore, vegans reported higher median (p25, p75) consumption of polyunsaturated fatty acids (PUFAs) (7.8 E%, 6.2, 10.3) than vegetarians (5.8 E%, 4.7, 8.0) (*P* = 0.011), and neither of the diet groups differed from pescatarians (6.7 E%, 5.1, 9.4) in relation to PUFA consumption (Table 2). A higher median (p25, p75) intake of n-3 fatty acids (1.2 E%, 0.7, 1.8) was reported amongst pescatarians compared with vegetarians (0.5 E%, 0.4, 1.0) (*P* < 0.001) and vegans (0.7 E%, 0.5, 1.2) (*P* = 0.010) (Table 2). No differences in monounsaturated fatty acid (MUFA) intake were observed between the groups (Table 2).

#### Protein, carbohydrates, added sugars and dietary fibre intake

All groups’ median (p25, p75) protein intake (E%) complied with NNR (Table 2). Pescatarians had the highest relative median (p25, p75) consumption of protein (14.9 E%, 13.0, 16.9) compared with vegans (13.3 E%, 11.5, 15.1) (*P* = 0.029) and vegetarians (13.2 E%, 10.3, 15.2) (*P* = 0.018). Carbohydrate intake did not differ between groups (Table 2). For all groups, the median (p25, p75) intake of added sugar was in line with recommendations from national health authorities (<10 E%), with a significantly higher intake amongst vegetarians (4.1 E%, 1.1, 8.4) compared with vegans (2.0 E%, 0.3, 4.9) (*P* = 0.014) and pescatarians (1.8 E%, 0.8, 4.6) (*P* = 0.046) (Table 2). Furthermore, our results indicated a higher median (p25, p75) intake of dietary fibre amongst vegans (5.2 g/MJ, 4.1, 6.0) compared with vegetarians (3.8 g/MJ, 2.8, 4.7) (*P* < 0.001) and pescatarians (4.2 g/MJ, 2.7, 5.1) (*P* = 0.005) (Table 2). Median fibre intake (g/MJ) complied with NNR in all groups.

#### Salt intake

Median salt intake (g) complied with NNR at levels equal to or lower than 6 g in vegans (6.0 g [3.6, 9.9]) and vegetarians (5.2 g [3.4, 8.1]) (Table 2). Pescatarians (7.0 [2.7, 5.1]) had a median salt intake above NNR recommendations.

### Consumption of plant-based substitutes

Altogether, 79.1% of the participants consumed either meat or dairy substitutes (Table 3). Consumption of substitute products differed between the diet groups (vegans, 90.4%; vegetarians, 68.1%; pescatarians, 64.3%; *P* = 0.001). Vegans had the highest percentage (80.7%) of participants reporting consumption of dairy substitutes, followed by vegetarians (57.4%) and pescatarians (50.0%) (*P* = 0.002). Consumption of meat substitutes alone did not differ significantly between the groups (*P* = 0.068).

**Table 3:** Percentages of participants reporting consumption of meat substitutes and/or dairy substitutes (*n* = 158)

Substitutes	Total		Vegans		Vegetarians		Pescatarians		*P*-value^1^
	*n*	%	*n*	%	*n*	%	*n*	%	
Meat substitutes	69	43.7	43^a^	51.8	18	38.3	8^a^	28.6	0.068
Dairy substitutes	108	68.4	67^ab^	80.7	27^a^	57.4	14^b^	50.0	0.002*
Both meat and dairy substitutes	52	32.9	35^a^	42.2	13	27.7	4^a^	14.3	0.017
Either meat or dairy substitutes	125	79.1	75^ab^	90.4	32^b^	68.1	18^a^	64.3	0.001

1 *P*-value for the difference between different diet groups, chi-square test, significance level used: < 0.05, marked as *.

ab Diet groups with the same superscripts have proportions that differ significantly in the post hoc test, significance level used < 0.05.

No differences were found between the groups in intake of different product categories of meat substitutes (data not shown). Consumption of dairy substitutes differed between diet groups in two product categories (data not shown). A higher percentage of vegans (69.9%) than vegetarians (44.7%) and pescatarians (42.9%) (*P* < 0.004) consumed milk substitutes. Furthermore, vegans (32.5%) had the highest percentage of participants reporting consumption of cheese substitutes (*P* = 0.005).

### Contribution of nutrients from meat and dairy substitutes to total dietary energy, macronutrient and salt intake

The contribution of energy and salt intake from meat and dairy substitutes to total energy and salt intake is presented in Table 4. The overall median (p25, p75) contribution of energy to total energy intake was 12.2% (6.9, 19.4), and no difference was found between the groups. Fat from substitute products contributed the most to total fat intake in vegans (19.0%, 8.4, 30.4) compared with pescatarians (10.0%, 4.3, 22.7) (*P* = 0.010), but no difference was observed between vegetarians and the other diet groups (Table 4). Moreover, SFAs from substitute products consumed by vegans contributed more to total SFA intake than substitute products consumed by vegetarians (26.7%, 10.3, 54.9) (*P* = 0.020) and pescatarians (7.6%, 1.9, 19.7) (*P* = 0.002) (Table 4). The contribution of trans fatty acids (TFAs), MUFAs, PUFAs, carbohydrates, added sugar, dietary fibre and salt from substitute products to total intake of these nutrients did not differ between the dietary groups (Table 4). However, overall vegans had higher median (p25, p75) protein intake from substitutes (19.1%, 6.2–36.9) than pescatarians (6.9%, 1.8, 17.1) (*P* = 0.03) (Table 4).

**Table 4:** Contribution from meat and dairy substitutes to total intake of energy (kcal), macronutrients (g) and salt (g) (*n* = 125)

Dietary factor	Total (*n* = 125)		Vegans (*n* = 75)		Vegetarians (*n* = 32)		Pescatarians (*n* = 18)		*P*-value^1^
	Median	(p25, p75)	Median	(p25, p75)	Median	(p25, p75)	Median	(p25, p75)	
Kcal%	12.2	(6.9, 19.4)	13.8	(8.1, 22.1)	10.3	(6.7, 16.5)	10.2	(2.3, 15.0)	0.061
Fat%	16.5	(8.4, 30.4)	19.0^a^	(10.4, 35.5)	13.7	(7.1, 23.2)	10.0^a^	(4.3, 22.7)	0.022*
SFA%	15.5	(5.5, 45.7)	26.7^ab^	(10.3, 54.9)	9.7^b^	(2.2, 44.6)	7.6^a^	(1.9, 19.7)	0.002*
TFA%	0.0	(0.0, 0.0)	0.0	(0.0, 0.0)	0.0	(0.0, 0.0)	0.0	(0.0, 0.0)	0.438
MUFA%	9.2	(4.2, 15.1)	9.5	(3.7, 16.0)	9.2	(5.3, 13.4)	7.6	(3.2, 15.9)	0.860
PUFA%	15.5	(5.2, 28.0)	14.7	(4.5, 27.9)	17.9	(8.2, 32.3)	13.8	(4.7, 26.4)	0.514
Protein%	14.2	(5.3, 31.4)	19.1^a^	(6.2, 36.9)	13.0	(6.1, 27.4)	6.9^a^	(1.8, 17.1)	0.011*
Carbohydrate%	7.9	(3.3, 12.8)	8.0	(3.2, 12.4)	7.4	(4.0, 16.4)	5.1	(1.6, 10.9)	0.372
Added sugar%	0.0	(0.0, 5.6)	0.0	(0.0, 5.9)	0.4	(0.0, 21.3)	0.0	(0.0, 0.4)	0.203
Dietary fibre%	9.9	(4.0, 17.1)	10.2	(4.1, 16.3)	7.1	(3.4, 19.6)	9.0	(1.7, 16.8)	0.671
Salt%	15.9	(5.1, 34.3)	17.7	(5.4, 41.2)	14.7	(6.0, 33.9)	7.1	(1.9, 21.7)	0.109

1 Tested for difference between different diet groups, Kruskal-Wallis test, significance level used: < 0.05, marked as *.

ab Diet groups with the same superscripts have proportions that differ significantly in the post hoc test, significance level used < 0.05.

### Raw ingredients

The main raw ingredient in the plant-based meat and dairy substitute products reported in the 24 h and the consumption frequency for these products are presented in Table 5. Amongst the meat substitutes, soy-based products were consumed most frequently, both in total (55.4%) and within each category: sausages (44.4%); burgers (55.6%); mince and ‘meatballs’ (53.8%); nuggets and schnitzel (63.6%) and other products (93.8%) (Table 5 and Supplementary Fig. 1). Peas were the main raw ingredient (20.5%) in all meat substitutes consumed: 50.0% of the cold cuts and spreads; 33.3% of the burgers; 15.4% of the mince and ‘meatballs’.

**Table 5:** Frequency percentage of meat and dairy substitute products reported in the 24 h based on the main raw ingredients

Meat substitutes		Dairy substitutes	
Raw ingredient	Frequency (%)^1^	Raw ingredient	Frequency (%)^2^
Soy	46 (55.4)	Oats	85 (35.0)
Peas	17 (20.5)	Soy	78 (32.1)
Legumes and vegetables	9 (10.8)	Modified Starch	39 (16.0)
Sunflower seeds	3 (3.6)	Coconut oil	8 (3.3)
Mycoprotein	2 (2.4)	Coconut milk	6 (2.5)
Other*	4 (4.8)	Almonds	7 (2.9)
Unspecified**	2 (2.4)	Cashew nuts	4 (1.6)
		Rice	5 (2.1)
		Other*	4 (1.6)
		Unspecified**	7 (2.9)

* Products with raw ingredients reported once.

** The specific product and, thus, the main ingredient could not be identified.

1 Total amount of products reported was 83.

2 Total amount of products reported was 243.

The dairy substitutes consumed most frequently were based on either oats (35.0%) or soy (32.1%) (Table 5). Within the milk-substitute category, oats and soy accounted for 46.5% and 42.4% of the products consumed, respectively. Whilst the yoghurts mostly were soy-based (60.9%), most creams and crème fraiche (66.7%) were made from oats (Supplementary Fig. 2). Modified starch was the main raw ingredient in 16.0% of all the dairy substitutes consumed and accounted for 79.6% of the consumed cheese substitutes (Table 5 and Supplementary Fig. 2).

## Discussion

This study provides a snapshot of meat and dairy substitute intake amongst people adhering to various plant-based diets. To sum up, the participants’ total macronutrient intake fell within NNR recommendations, presenting a favourable distribution of fatty acids in addition to high levels of dietary fibre. Most participants had consumed meat or dairy substitutes, and the consumption was most frequent amongst vegans, followed by vegetarians and pescatarians. Vegans’ consumption of substitutes led to higher intake of total fat, SFAs and protein compared with the other groups.

Whilst traditional plant-based diets have focussed on whole foods, the modern adaptation of these diets may include highly processed meat and dairy alternatives (23). Little is known about plant-based substitutes’ impact on diet quality. Recent studies suggest that meat and dairy substitutes are associated with higher intake of ultra-processed foods and less healthy eating patterns in plant-based diets (23, 36). However, studies have also found meat substitutes contain less total and saturated fat, and more dietary fibre than their meat counterparts, although some products contained more sodium (15, 37). Gibney raised the question of whether the consumption of processed foods, for example, meat and dairy substitutes, matter if total nutrient intake remains within recommendations for optimal nutrient intake (38).

### Macronutrients

All groups reported total macronutrient intake within NNR 2012 (35), and contrasts in macronutrient intake usually were the strongest between vegans and pescatarians, except for dietary fibre and added sugar. Similar to our results, other studies have found that vegans have a more favourable distribution of macronutrients, with a lower contribution by SFAs (9, 39) and a higher contribution of PUFAs (9, 40) to total energy intake, compared with vegetarians and pescatarians. These findings were as expected because SFAs mostly are found in animal-based foods, for example, whole-fat dairy products. Therefore, vegans have few natural SFA sources in their diets. Moreover, vegan diets may include ample amounts of plant-based oils, nuts, seeds and whole grains, which are good PUFA sources (7, 13). Pescatarians were the only group to meet the requirement of ≥1 E% n-3 fatty acids in the present study (35), which most likely may be explained by regular consumption of fatty fish. However, low intake of n-3 fatty acids amongst vegans (0.7 E%) and vegetarians (0.5 E%) most likely may indicate that they do not include an adequate amount of n-3-rich plant-based food sources (e.g. flaxseeds and flax seed oil, chia seeds and walnuts) or supplements to meet their requirements.

An analysis of protein intake in the European Prospective Investigation into Cancer and Nutrition-Oxford study (EPIC-Oxford study) found a rising gradient, with the lowest intakes in vegans, followed by vegetarians and the highest in pescatarians (40). A similar gradient also was observed in the French NutriNet-Santé study, although that study did not distinguish between vegetarians and pescatarians (7). Even though median intakes of protein amongst vegans (13 E%) and pescatarians (15 E%) in the present study were almost equal to findings from the EPIC-Oxford study, vegetarians in the present study reported protein intakes similar to those of vegans, thereby deviating from the previously observed gradient (40). However, the protein intake was in line with the NNR in all the dietary groups.

Previous studies have found that dietary fibre intake increases when the intake of animal source food decreases, with the highest levels observed in vegans compared with other diets (7, 8, 39, 40, 41). Similarly, in the present study, vegans reported the highest intake of dietary fibre in g/MJ, whereas no difference was observed between vegetarians and pescatarians. However, the median intake of dietary fibre in all three groups (5.2, 3.8 and 4.2 g/MJ) exceeded the estimated average intake in the general Norwegian population (2.3 g/MJ) (42). The high intake of dietary fibre reported amongst all groups included in the present study was likely a result of the high consumption of whole grains, legumes, vegetables, fruits, nuts and seeds. These food groups represent the main components of all healthy plant-based diets (13) and often are consumed in large amounts by vegans, vegetarians and pescatarians compared with omnivores (7, 39, 41, 43).

Although vegetarians in the present study reported a higher intake of added sugar than the other two groups, the intake in all groups was within the recommendations of <10 E% and below the estimated average intake in the general Norwegian population (11 E%) (42). A possible explanation for this low intake of added sugar may be that the study’s participants are more conscious about their health and have more nutritional knowledge compared with the general population. However, underreporting attributed to social-desirability bias cannot be ruled out. Participants, inadvertently or deliberately, may have neglected to mention the intake of sugary foods in the 24 h or underestimated the amount eaten.

Salt intake reported in this study was considerably lower than the estimated average salt intake in the Norwegian population (10 g/day) (44). However, only vegans (6.0 g) and vegetarians (5.2 g) met NNR recommendations (35) of ≤6 g/day. The salt intake levels reported in this study concurred with several studies that found vegans and vegetarians consumed less sodium than meat-eaters (7, 41, 45). However, both the EPIC-Oxford study (40) and Adventist Health Study 2 (8) found no difference in sodium intakes amongst meat-eaters, pescatarians, vegetarians and vegans.

### Consumption of plant-based meat and dairy substitutes

Plant-based meat and dairy substitutes have been flooding the global market, and replacing regular meat or dairy with these products, rather than whole foods, has become increasingly common (20). These trends were reflected in the results presented in our study, as most participants reported consuming either meat or dairy substitutes during the previous 24 h. Previous studies have suggested that the consumption of substitute products rises with increased avoidance of animal-source foods (7, 23, 43, 46). Supporting this hypothesis and corresponding with all groups avoiding meat, the present study found no differences between groups in consumption of each product category of meat substitutes. Furthermore, our results found that the most prominent differences in dairy substitutes’ consumption patterns lied in the milk and cheese categories. Whilst cow’s milk and cheese, often consumed daily in the Norwegian diet, are included in vegetarian and pescatarian diets, vegans would need to replace these products with plant-based substitutes. This study’s findings suggest that plant-based substitutes for both meat and dairy may provide a convenient way of maintaining food habits by replacing foods and beverages excluded from different eating patterns.

Another potential explanation for the large proportion of vegans who reported consuming substitutes is that these products are viewed as good sources of protein (soy-based meat substitutes) or micronutrients (fortified dairy substitutes). Considering that soybeans were the main ingredient in early meat substitutes, for example, tofu and tempeh, these have long been recognised as a source of high-quality protein in vegetarians’ diets (1, 46, 47). Furthermore, soybeans have been recommended as a source of iron, potassium, zinc and selenium in diets that exclude meat (1, 46, 47). More than 55% of the meat substitutes and 32% of the dairy substitutes reported as being consumed in this study were soy-based, suggesting that such products are chosen frequently. However, most dairy substitutes consumed were oat-based, probably due to the popularity and wide range of oat-based dairy substitute products, as well as oats’ prominence in the Norwegian diet. Many variants of plant-based substitutes for milk on the Norwegian market are fortified with calcium, vitamin B_12_ and vitamin D, and a recent report from the Norwegian National Nutrition Council has recommended including calcium-fortified, plant-based substitutes for milk from soy as an alternative to cow’s milk (48). Considering that most participants in the present study had followed their current diet for several years, they likely were well-informed about how to compose a nutritionally adequate diet and consequently may have included substitute products to ensure intake of certain nutrients. Although soy or oats were the preferred raw ingredients in most dairy substitute categories, 80% of the plant-based cheese substitutes comprised modified starches. Consistent with this observation, a recent study on the nutritional composition and quality of plant-based cheese found that most products comprised a combination of refined coconut oil and starches, with refined coconut nut oil being the main ingredient (49).

### Contribution to macronutrient and salt intake from plant-based meat and dairy substitutes

Amongst participants who reported consuming plant-based substitutes in our study, vegans reported the highest contribution of total fat, SFAs and protein from substitute products. Considering that our results indicated no differences in total energy intake (kcal) or total fat intake (E%) between the groups, the greater contribution from substitutes to total fat in vegans compared with pescatarians may be explained by higher consumption of these products amongst vegans compared with pescatarians. However, plant-based substitutes’ nutrient content has been found to vary greatly (15, 17, 22, 49, 50), and although not assessed in this study, it is possible that products that vegans consumed frequently contained more fat than the products that pescatarians consumed. The prominent difference in consumption of dairy substitutes found between the diet groups may be one possible explanation for differences in contributions from substitutes to SFA intake. However, except for coconut-based milk, milk substitutes tend to have a lower SFA content than cow’s milk (19, 50, 51). Furthermore, few participants reported having consumed coconut-based milk substitutes, making it unlikely that the consumption of plant-based milk substitutes could explain the difference in SFA intake between the diet groups. A more plausible explanation is that whilst vegetarians and pescatarians may consume whole-fat dairy containing significant SFA amounts, vegans have few natural SFA sources in their diets. Thus, SFAs from substitutes likely account for a larger proportion of total SFA intake amongst vegans than amongst vegetarians and pescatarians.

Similarly, vegans’ higher protein intake from consuming substitutes (19%) compared with pescatarians (19 and 7%, respectively) also may be explained by the lower total protein intake observed in vegans compared with pescatarians in this study. However, Bradbury et al. (25) also found that the consumption of vegetarian protein alternatives (excluding legumes, nuts and seeds) and plant-based substitutes for milk was higher amongst vegans than pescatarians, with vegetarians reporting intermediate values.

Although substitutes’ contribution to total salt intake did not differ between groups, the median contribution to total salt intake was substantial in vegans (18%) and vegetarians (15%), suggesting a potential negative impact on salt intake from substitute products. Plant-based substitutes for neither milk nor cheese have been found to contain high levels of sodium (49, 51). However, whether due to requirements in processing or efforts to improve the product’s taste, high sodium content is one of the main concerns regarding meat substitutes’ nutrient content (15, 22).

### Strengths and limitations

The strength of this study is that the 24 h were conducted as personal interviews, instead of online surveys, thereby providing data with high levels of detail. Furthermore, the same researcher conducted all interviews, thereby eliminating researcher bias. Considering that the participants were not informed in advance that they would be asked to account for their dietary intake in detail, they were unable to adjust their diets, which increased the likelihood of recalled intake representing true normal intake. Furthermore, information was logged consecutively in written records, and calculations were performed in detail to increase data accuracy.

One of the main limitations of this study was the use of single 24 h dietary recall per person, as it does not provide information on habitual intake (52). Combined with the relatively small sample especially in the group of pescatarians, the results should be interpreted with caution. Using convenience and snowball sampling methods may have introduced self-selection bias and weakened the findings’ generalisability. Participants mostly were from urban areas of Norway, near Oslo, the capital, and had higher education levels; thus, the sample may not be representative of vegans, vegetarians and pescatarians living in other parts of the country with lower education levels. However, plant-based diets are likely more common amongst people living in Oslo (20%), compared with the country in general (10%) (4), and higher education is associated positively with plant-based eating (53), thereby strengthening the sample’s representativeness.

Because fortified plant-based substitutes have been suggested as contributing substantially to the intake of several micronutrients of concern in plant-based diets (8), another limitation of this study is that only macronutrient intake was assessed. Unfortunately, due to a lack of information on the nutrient content of meat and dairy substitute products not registered in the Norwegian Food Composition Table, the data were insufficient to analyse plant-based substitutes’ contribution to total intake of micronutrients and n-3 fatty acids. Further research is needed to evaluate fortified plant-based substitutes’ contribution to micronutrient intake – for example, calcium, iodine, B_12_ and vitamin D – in Norwegian plant-based diets.

## Conclusion

To sum up, all groups reported a total macronutrient intake within NNR recommendations, presenting a favourable intake of fatty acids and dietary fibre based on the recommendations. Most participants consumed meat or dairy substitute products, suggesting that these products are included regularly in Norwegian plant-based diets. Consuming plant-based substitutes was reported most frequently by vegans, followed by vegetarians and pescatarians. Our results indicated a higher contribution from substitute products to intake of total fat, SFAs and protein amongst vegans compared with the other groups. However, more studies are needed to gain knowledge about plant-based substitutes’ diet and health effects, and these studies should assess both macro- and micronutrients in larger samples.
