# Supplementary material for: Consumption of meat and dairy substitute products amongst vegans, vegetarians and pescatarians
Source: Food Nutr Res. 2023 Mar 31;67:10.29219/fnr.v67.9081. doi: 10.29219/fnr.v67.9081 (PMC10084502; doi:10.29219/fnr.v67.9081)
Supplement: Supplementary file 1 [file FNR-67-9081-s001.docx]

Supplementary materials

### Figure 1. Proportions within each category of all meat substitute products reported in 24h by 125 participants based on raw ingredients and intake frequency. ‘Other’ products are based on raw ingredients reported only once.‘Unspecified’ includes unidentifiable products and raw ingredients.

### Figure 2. Proportions within each category of all dairy substitute products reported in 24h by 125 participants based on raw ingredients and intake frequency. The category ‘Other’ includes products based on raw ingredients reported only once. The category ‘Unspecified’ includes unidentifiable products and raw ingredients.
